# Study of the effect of the essential oil (extract) of rhubarb stem (shoot) on glycosylated hemoglobin and fasting blood glucose levels in patients with type II diabetes

**DOI:** 10.1051/bmdcn/2018080424

**Published:** 2018-11-26

**Authors:** Fahimeh Shojaei Shad, Maryam Jahantigh Haghighi

**Affiliations:** 1 Department of Nursing, Faculty of Nursing and Midwifery, Zabol University of Medical Sciences Zabol Iran

**Keywords:** Rhubarb, Glycosylated hemoglobin, Fasting blood glucose, Type II diabetes

## Abstract

Background: Diabetes is a serious chronic disease that can damage the heart, arteries, eyes, kidneys and nerves, leading to death and early disability. Before the discovery of insulin as well as common anti-diabetes drugs, patients with diabetes were treated with medicinal herbs and traditional treatments. One of these effective medicinal herbs is Rhubarb. Rhubarb is prescribed in traditional medicine for various patients, including patients with diabetes. But its effect has not been scientifically reported so far.

Purpose: This study was conducted with the aim to determine the effectiveness of Rhubarb stem extract on HbA1C and fasting blood glucose in patients with type II diabetes.

Method: In this experimental study, 80 patients with type II diabetes mellitus in Zabol diabetes center, aged 30-60 years old with fasting blood glucose greater than 140 mg/d/ were selected. Patients were randomly assigned into two groups (n = 40) of treatment with capsules of Rhubarb stem and placebo after matching the oral medications. The patients in both groups were studied for fasting blood glucose and HbA1C before and after 1 month of conducting the study.

Results: The mean FBS and HbA1C in rhubarb group before the intervention were 288.80 ± 94.49 and 9.62 ± 1.58, respectively, and after the intervention were 226.42 ± 88.89 and 7.83 ± 1.50, respectively. According to the statistical paired f-test and Wilcoxon test a significant reduction was found in FBS and HbA1C with rhubarb intervention *(p* < 0.05).

Conclusion: In diabetes mellitus, as many factors affect the level of blood glucose, they also contribute to reducing blood glucose level and preventing complications. Therefore, considering the positive effects of rhubarb, it can be recommended to use rhubarb extract as an additional treatment to reduce blood glucose level.

## 1. Introduction

Today, diabetes is known as a major health problem and medical emergency in the world due to its high prevalence [1]. The global outbreak of diabetes in 2017 was more than 425 persons worldwide which, with an increase of 48% will reach over 629 million persons by 2045. In the Eastern Mediterranean countries in 2017, more than 39 million people were diagnosed with diabetes, and is expected to reach over 82 million in 2045. Developing countries, such as those in the Eastern Mediterranean including Iran, have the highest rate of diabetes worldwide so that among every 5 persons with diabetes, 4 persons live in these countries. The annual prevalence of diabetes in Iran is 9.6% [1, 2].

**Table 1:** Comparison of mean FBS and HbA1C, systolic and diastolic blood pressure before and after the intervention in the placebo group.

diastolic lood pressure	systolic blood pressure	HbA1C	FBS	
95.37 ± 8.72	145.50 ± 9.04	9.52 ± 1.67	252.55 ± 82.98	Before the interventio
93.37 ± 7.95	143.75 ± 11.91	9.49 ± 1.68	252.72 ± 85.33	After the intervention
0.19	0.31	0.72	0.94	*p*-value

**Table 2:** Comparison of mean FBS and HbA1C, systolic and diastolic blood pressure before and after the intervention in rhubarb group.

diastolic blood pressure	systolic blood pressure	HbA1C	FBS	
95.75 ± 7.72	144.87 ± 8.73	9.62 ± 1.58	288.8 ± 94.49	Before the intervention
88.25 ± 8.73	133.50 ± 10.98	7.83 ± 1.5	226.42 ± 88.89	After the intervention
0.0001	0.0001	0.0001	0.0001	*p*-value

Diabetes mellitus is characterized by symptoms such as hyperglycemia, polyuria, polydipsia, weight loss, delayed healing of the ulcers, blurred vision, increased glucose in the urine and some other symptoms [9]. The importance of this disease is due to the prevalence of its complications. Diabetes can damage the heart, arteries, eyes, kidneys and nerves, leading to death and early disability [1, 10]. Diabetes complications include skin lesions, hypertension and weight gain [11], also vascular complications of diabetes include neuropathy, nephropathy and retinopathy and macro-vascular diseases, which are among the leading causes of death in diabetic patients. Blood glucose as well as high blood pressure and high blood fat contribute to the risk of cardiovascular diseases [11].

Hemoglobin A is the main hemoglobin in adults which, when it binds to glucose is called A1. Glycosylated hemoglobin (HbA1C) is a major component of HbA1 and forms 4-6% of hemoglobin in healthy individuals [12]. Measuring glycosylated hemoglobin is a standard method for long-term blood glucose control. Study glycosylated hemoglobin is more important in comparison with fasting blood glucose in order to diagnose diabetes [13]. The amount of glycosylated hemoglobin represents the mean blood glucose over the past few weeks [1].

The number of patients with diabetes is rapidly increasing due to the population growth, population aging, urbanization and industrialization, and an increase in the prevalence of obesity and physical inactivity [14]. Type II diabetes is associated with an increase in mortality and a decline in the quality of life. Therefore, it imposes much more economic burden on the health systems and community [15].

In order to control diabetes, changes in the lifestyle need to be made. The evidence suggests that if you have adequate blood glucose control, you can prevent or delay the long-term complications of diabetes [16]. The primary treatment of type II diabetes is oral anti-diabetic drugs [17]. These drugs have disadvantages, such as drug resistance development, side effects, even toxicity and lack of response. Moreover, none of these glucose lowering drugs effectively control the increase in blood fat. Therefore, with the increasing prevalence of diabetes and the adverse impacts of synthetic drugs, there is a clear need for the development of natural herbal resources for anti-diabetic drugs [11]. Rhubarb with the scientific name of Rheum Ribes is a perennial plant belonging to the Polygonaceae family [18], known as Rhubarb. Rhubarb root is used to treat diabetes [18, 19], hypertension, ulcers, obesity, diarrhea, and its mucolytic agents [20]. Rhubarb stem has a laxative effect and is used to treat constipation. Its root extract is also used to treat diabetes, stomach, liver, smallpox and rubella. Antioxidant properties and the presence of flavonoids in rhubarb, including Quercetin, have been effective on blood glucose level and metabolic disorders in diabetes [21]. One of the most important properties of rhubarb is that it causes the pancreas to secrete insulin and hence reduces blood glucose level [22, 23]. Rhubarb reduces blood fat and pressure and, in combination with cinnamon, has anti-fat and diabetes effects. The study of Fallah Hosseini (2007) showed that a significant relationship was found between the use of rhubarb and the reduction of fasting blood glucose, total cholesterol and LDL levels in type II diabetic patients [24]. Hamza (2014) studied the effect of rhubarb on histopathologic changes in pancreas and blood glucose in diabetic patients and the results of the study indicated that rhubarb had hypoglycemic effects and was effective on the treatment of type II diabetes [21]. Adham’s (2015) study also showed that rhubarb root reduced the level of diabetes in diabetic patients (18). Given that today the community of doctors and nurses has concluded that nurses can play a fundamental role in the treatment and care of diabetes mellitus and its complications [25], and due to the increased prevalence of diabetes and its mortality, this study was conducted to determine the effectiveness of Rhubarb stem extract on HbA1C and fasting blood glucose levels in type II diabetic patients.

## 2. Method

This study was an experimental study and was performed on 80 patients with type II diabetes mellitus in Zabol, Iran in 2017. After matching the oral medications, the subjects were randomly divided into two groups of 40 patients receiving rhubarb extract and placebo. The inclusion criteria of the study included aged 35-60 years, having type II diabetes for at least 6 months, fasting blood sugar of more than 140 mg/dl for at least two trials, ability to speak, ability to move and do activity, and lack of delayed complications of diabetes such as cardiovascular disease, foot ulcer, eye problems according to the physician, lack of Alzheimer’s disease or mental diseases, lack of pregnancy or lactation, and no autoimmune disorder [7, 26]. The exclusion criteria included having severe physical diseases, changed drug therapy in controlling diabetes from oral blood glucose lowering medications to insulin therapy or dietary changes, unwillingness to use capsules, unwillingness to participate in the study, smoking, alcohol and drug abuse. After explaining the purpose of the study for the patients and obtaining informed written consent, a general information questionnaire was completed including demographic information [27] and information about the disease through interviewing each patient. Then, for both groups, HbA1C and fasting blood glucose were measured. All patients were urged to continue taking the blood glucose lowering medications without any changes to the treatment regime and diet. Packages of 90 capsules of medication and placebo were similar in appearance, specified by a special code, delivered to the patients by the therapist without any knowledge of it, and a special code was inserted in the patient’s file. The rhubarb was made from Binalod Mountain in Neishabur city, and approved by the Department of Biology, Zabul University of Medical Sciences. At first, the plant was powdered with an electric mill, after which it was dried, and in the final stage, the powder was prepared as 400 mg capsules. The patients in the intervention group received a bottle of 90 capsules of 400 mg (3 tablets daily: morning, noon, and night) of the stem extract of rhubarb, and the placebo group’s patients, similarly, a bottle of 90 placebo capsules of 400 mg. The patients in both groups were studied for HbA1C and fasting blood glucose levels at the end of the study after one month. All patients participated from start to end of study. This study was approved by the ethics committee of Zabol University of Medical Sciences. The analytical tests were used for data analysis.

## 3. Results

The results of this study showed that the mean age of subjects in the intervention group was 50.22 ± 6.72 and 50.57 ± 6.60 in the control group (placebo). The mean weight before the study in the intervention group was 74.97 ± 10.98 and 75.85 ± 12.19 in the control group. 27.5% of the participants in both control and intervention groups were male (n = 11) and 72.5% were female (n = 29). Most of the subjects were illiterate in both control (65%) and intervention (72.5%) groups. No significant difference was found between the subjects in the intervention and control groups in terms of demographic factors *(p* < 0.05). No significant difference was found between the two intervention and control groups regarding the levels of FBS and jHbAlC according to the statistical independent f-test and Mann-Whitney test (*p* <0.05).

The mean FBS in the intervention group (rhubarb) before the intervention was 288.80 ± 94.49 and 226.42 ± 88.89 after the intervention. According to the statistical paired f-test, this reduction in fasting blood glucose after rhubarb intervention was significant with *p* > 0.05.

The mean FBS in the control group (placebo) was 252.55 ± 82.98 before placebo, which increased by 252.72 ± 85.33 after using the placebo. No significant difference was found between fasting blood glucose before and after placebo (p = 0.94) according to the statistical paired *t*-test.

The mean HbAlC in the rhubarb intervention group before the intervention was 9.62 ± 1.58 which reduced to 7.83 ± 1.50 after the intervention. According to the statistical paired f-test, this reduction was significant *(p* < 0.05).

The mean HbAlC in the placebo group before the intervention was 9.25 ± 1.67 that reduced to 9.49 ± 1.68 after the intervention. According to the statistical paired f-test, this reduction was not significant in HbA1C *(p* = 0.72).

## 4. Discussion

The present study suggests that the mean FBS and HbA1C in placebo group, before and after using placebo, did not change significantly according to the statistical paired f-test. The mean HbA1C was reduced after rhubarb use. According to the statistical paired f-test, this reduction in HbA1C was significant with rhubarb use. Also, according to the results of the present study, FBS level before the intervention in rhubarb group was 288/80 ± 94/49 and 226.42 ± 88.89 after taking 3-month oral capsules of rhubarb stems, which indicates a significant reduction in fasting blood glucose level with taking Rhubarb stem capsule in type II diabetic patients. This is consistent with the study of Goel *ef al.* (1997). Goel *ef al.* attributed the effect of reducing blood glucose level of rhubarb to tannin, they showed that tannin stimulates pancreatic beta cells. The effect of rhubarb on beta cell activity causes a reduction in blood glucose level, which helps regulate it [28]. Fallah Hosseini *ef al.* (2008) reported that daily consumption of 1200 mg rhubarb stem extract by diabetic patients with high fat significantly reduced total cholesterol, LDL, triglyceride and blood glucose levels, which was consistent with our study. Also, in the study of Fallah Hosseini, it was found that this herbal drug had no effect on the level of liver enzymes SGOT, SGPT, and creatinine, indicating the health and safety of rhubarb.[24] In the present study, also none of the subjects reported any complications during taking the oral rhubarb capsules. AL-Bayaty *ef al.* (2006) in a study entitled “study the effect of Rhubarb stem extract on diabetic rabbits with Alloxan” also showed that the use of Rhubarb stem extract significantly reduced blood glucose level [29], which is consistent with our study. According to Tosun (2003) study, Rhubarb stem had flavonoids, including Quercetin with a significant concentration [30]. Nuraliev *ef al.* (1992) in their study entitled Quercetin hypoglycemia effect on diabetic rats with Alloxan” also showed that Quercetin in rhubarb significantly reduced LDL, glucose and cholesterol levels [31] which is consistent with our study. Studies by Skottova (1998) and Wang (2000) suggested that the effects of rhubarb on reducing blood fat and glucose were associated with its antioxidant properties [32, 33] which are consistent with our study in terms of the effects of rhubarb on reducing blood glucose levels. The study by Hamza *ef al.* (2014) entitled “the effect of hydroalcoholic extract of root of rhizobacteria and histopathologic changes of pancreas on diabetic rats with Alloxan” showed that the levels of glucose, cholesterol, triglyceride and weight gain in rats treated with Rhubarb root extract had a significant reduction compared to the diabetic control group [21] which is consistent with our study on the effects of Rhubarb on reducing blood glucose level with the difference being that we used Rhubarb stems in our study. Adham and Naq- ishbandi (2015) in a study also found that the patients treated with rhubarb root after the 12^th^ week showed a significant reduction of 39.63% at the glucose level [20] which is consistent with our study, the only difference being that Adham *ef al*. used the root of the rhubarb. Also, Rashidi *ef* al.’s (2013) study entitled “review the herbs used for diabetes mellitus” showed that one of the best herbs in controlling hyperglycemia is rhubarb [34]. The study by Hamza and Fallah also emphasized the antioxidant effects of this plant [20, 23]. Anti-oxidant properties and the presence of flavonoids in rhubarb, including Quercetin, are effective on blood glucose and metabolic disorders in diabetic patients. Also, the results of studies have shown that using different doses of rhizome powder in rhubarb reduces blood glucose levels [35–37].

## 5. Conclusion

Since blood glucose control can prevent serious complications of diabetes, so the present study, considering the effects of Rhubarb stem on reducing FBS and HbA1C in diabetic patients, suggests that herbal medicines, especially rhubarb should be considered in the treatment plan for these patients and extensive research should be conducted with a larger sample size in relation to the effects of this herb on type II diabetic patients.

## Conflict of interest statement

The authors disclose no conflicts of interest.
